# Improved High-Throughput Sequencing of the Human Oral Microbiome: From Illumina to PacBio

**DOI:** 10.1155/2020/6678872

**Published:** 2020-12-11

**Authors:** Jie Zhang, Lingkai Su, Yuan Wang, Shuli Deng

**Affiliations:** The Affiliated Hospital of Stomatology, School of Stomatology, Zhejiang University School of Medicine and Key Laboratory of Oral Biomedical Research of Zhejiang Province, Hangzhou, Zhejiang 310006, China

## Abstract

**Background:**

A comprehensive understanding of the commensal microflora and its relation to health is essential for preventing and combating diseases. The aim of this study was to examine the structure of the oral microbiome by using different sequencing technologies. *Material and Methods*. Five preschool children with no symptoms of oral and systemic diseases were recruited. Samples of saliva were collected. A 468 bp insert size library was constructed on the MiSeq platform and then subjected to 300 bp paired-end sequencing. Libraries with longer insert sizes, including a full-length 16S rDNA gene, were sequenced on the PacBio RS II platform.

**Results:**

A total of 122.6 Mb of raw data, including 244,967 high-quality sequences, were generated by the MiSeq platform, while 134.6 Mb of raw data, including 70,030 high-quality reads, were generated by the PacBio RS II platform. Clustering of the unique sequences into OTUs at 3% dissimilarity resulted in an average of 225 OTUs on the MiSeq platform; however, the number of OTUs generated on the PacBio RS II platform was 449, far greater than the number of OTUs generated on the MiSeq platform. A total of 437 species belonging to 10 phyla and 60 genera were detected by the PacBio RS II platform, while 163 species belonging to 12 phyla and 72 genera were detected by the MiSeq platform.

**Conclusions:**

The oral microflora of healthy Chinese children were analyzed. Compared with traditional 16S rRNA sequencing technology, the PacBio system, despite providing a lower amount of clean data, surpassed the resolution of the MiSeq platform by improving the read length and annotating the nucleotide sequences at the species or strain level. This trial is registered with NCT02341352.

## 1. Introduction

The human oral microbiome comprises over 700 prevalent taxa at the species level, including a large number of opportunistic pathogens involved in periodontal, respiratory, cardiovascular, and systemic diseases [1–5]. Identification of oral microorganisms at the species level is the basis and prerequisite for analyzing microbial communities of the oral cavity. The 16S rRNA gene is considered the gold standard for phylogenetic studies of microbial communities and high-throughput sequencing of the 16S rRNA gene could provide snapshots of microbial communities, revealing phylogeny and the abundances of microbial populations across diverse ecosystems [6, 7]. For this reason, the sequencing techniques had become an important tool for understanding the biology and functional characterization of oral microorganisms.

The emergence of the next-generation sequencers (NGS) and their sequencing by synthesis have drastically transformed the way scientists delve into the relationship between microbiome and related diseases [8]. Since then, many studies have used the NGS technologies, such as Roche/454 [9], ABI/Solid, Illumina [10], and its upgrade platforms including Illumina/HiSeq and MiSeq for microbial ecosystem analysis [9–15]. When it comes to the resolution and accuracy of the sequencing results, lengths and quantity of reads are very important factors [16–18]. Unfortunately, the NGS came with this drawback. Compared with the previous methods (e.g., Sanger sequencing), the reads generated are short. This became a major challenge for the assembly, especially in the case of large repetitive genomes [19]. Thus, in spite of the low cost and extremely high-throughput, the NGS platform is sometimes less accurate as a result of short read lengths and long repeats present in multiple copies [17]. Besides, although the explosion of sequence data brought about by high-throughput sequencing technologies is highlighting a richness of microbes not previously anticipated, not all of the novel organisms discovered by the NGS can be named by taxonomists because the existing tools are not sufficient to provide species names or phylogenetic information for the millions of short reads [20]. Operational taxonomic units (OTUs) at the 97% similarity is recognized as providing differentiation of bacterial organisms below the genus level [12]; however, it was still inaccurate for the reason that this level of clustering defines either microbial species or strains.

Third-generation sequencing (TGS), PacBio single molecule, real-time (SMRT) sequencing technology circumvented this problem by greatly increasing read lengths that have the ability to sequence the full length of the 16S rRNA gene [16, 18]. It involves a DNA fragment sequenced by a single DNA polymerase molecule connected to the bottom of a zero-mode waveguide [18]. During DNA synthesis, each of the nucleotides is illuminated upon incorporation, which can enable for identification. The PacBio RS II can yield average sequence reads of greater than 2500 bp; however, some research data show that circular consensus sequencing (CCS) of shorter fragments (<1500 bp) can decrease the sequencing errors [21]. Some studies have shown that the longer reads generated from sequencing the entire 16S rRNA gene provide a higher resolution of organisms and higher estimates of richness [17]. A previous study has shown that PacBio outperformed the other sequencers such as Roche 454 and MiSeq in terms of the length of contigs and reconstructed the greatest portion of the genome when sequencing the genome of *Vibrio parahaemolyticus* [22]. However, there have been few studies that aim at comparing the next-generation sequencing technology with PacBio RS II in oral microbiome. In this study, we explore the microbiota of oral cavity using sequences amplified V3-V4 and the V1-V9 small subunit ribosomal RNA (16S) hypervariable regions by two different platforms. The aim of this study was to evaluate the performance of TGS technology PacBio RS II in comparison with NGS technology Illumina/MiSeq for the structure of oral microbiome in 5 healthy preschool children in China.

## 2. Materials and Methods

### 2.1. Patient Information

Five preschool children aged 63–74 months, lacking evidence of oral and systematic diseases were recruited based on a list of exclusion criteria on Nov 26, 2014. The subjects with a history of chronic antibiotic used within 8 weeks before enrollment were excluded from the study. All subjects' legally authorized representatives provided written informed consent upon enrollment. The study was approved by the Institutional Review Board of the Affiliated Stomatology Hospital of Zhejiang University in accordance with the Declaration of Helsinki principles.

### 2.2. Saliva Sampling and Isolation of Bacterial DNAs

The subjects were instructed neither to eat and drink nor to perform any oral hygiene procedure two hours before sampling. Saliva samples were collected from all subjects in the morning between 9 : 00 am and 11 : 00 am.

Unstimulated saliva samples were collected according to a protocol, modified from a previous study. The children were initially asked to rinse their mouth thoroughly with deionized water prior to sampling, followed by collection of at least 5 mL unstimulated saliva in a plastic cup. Finally, the samples were transferred into sterile cryogenic vials. Then, the samples were placed into liquid nitrogen and stored at −80°C until use.

Bacterial DNAs were extracted using the E.Z.N.A.™ Soil DNA Kit (Qiagen, Omega, USA), according to the instructions of the manufacturer. The enriched microbial DNAs were purified by ethanol precipitation. DNA concentration was measured using NanoDrop, and its molecular size was estimated by agarose gel electrophoresis. DNAs were stored at −20°C until use.

### 2.3. PCR Amplification of the 16S rRNA Gene

PCR amplification of the 16S rRNA gene hypervariable V3-V4 regions was performed with universal bacterial primers 338F (5′-ACTCCTACGGGAGGCAGCA-3′) and 806R (5′-GGACTACHVGGGTWTCTAAT-3′). The V1-V9 hypervariable region was performed with primers 27F (5′-AGAGTTTGATCCTGGCTCAG-3′) and 1492R (5′-GGTTACCTTGTTACGACTT-3′). The products were extracted with the AxyPrep DNA Gel Extraction kit (Qiagen, USA) and were then examined by agarose gel electrophoresis. According to the electrophoretic results, the PCR products were quantified by Quantifluo™-ST (Promega, USA). Then, the products from different samples were then mixed at equal ratios for pyrosequencing on the two different platforms.

### 2.4. DNA Library Construction and Sequencing

**Figure 1:**
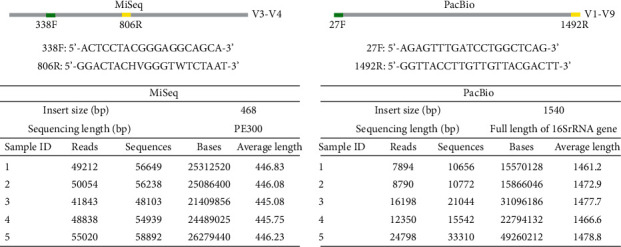
Sequencing results from 5 oral samples. Partial 16S amplicons (V3-V4) were sequenced on the Illumina/MiSeq; and full-length 16S amplicons (V1-V9) were sequenced on PacBio.

Construction of DNA library was carried out by following the manufacturer's instructions (Illumina and PacBio). A 468 bp insert size library was constructed on the MiSeq platform and then applied to 300 bp paired-end sequencing. Libraries with longer insert size (1540 bp) were performed on the PacBio RS II platform, including full length of 16S rDNA gene. Barcoded 16S rRNA amplicons (V3-V4 and V1-V9 hypervariable regions) of the five Chinese children were sequenced on MiSeq and PacBio RS II platforms, respectively. Raw data were generated, and low-quality reads were then removed by quality control (Figure 1).

### 2.5. Bioinformatic Analysis

We used QIIME software to cluster filtered reads into operational taxonomic units (OTUs) from PacBio and MiSeq platforms [23] by applying a 97% identity threshold relative to a centroid sequence. The generated OTUs were used for alpha-diversity (Shannon and Simpson), richness (Chao, ACE), coverage, and rarefaction curves using Mothur software (version v.1.30.1) [24]. We then assigned the resulting OTUs using a BLAST-based method implemented in QIIME, employing the SILVA (version 119) database as the reference for taxonomic analysis [25, 26]. The species-level operational taxonomic units (OTUs) and relative richness of phylum, class, order, family, genus, and species for each sample between the two platforms were compared. Statistical analysis was performed using SPSS for Windows (version 19.0; SPSS Inc., Chicago, IL, USA).

## 3. Results

### 3.1. Increased Diversity of Oral Microbiota Sequenced by TGS

By high-throughput pyrosequencing of 5 samples synchronously on two different platforms, a total of 122.6 Mb raw data including 244,967 high-quality sequences were generated by the MiSeq platform, while 134.6 Mb raw data including 70,030 high-quality reads were generated by the PacBio RS II platform. For the MiSeq platform, 99.99% of the clean reads distribution ranged from 401 to 500 bp, and for the PacBio RS II platform, 94.24% of the clean reads were distributed from 1401 to 1600 bp.

The average lengths of quality reads were 446 bp and 1471 bp on MiSeq and PacBio RS II platforms, respectively. With accurate read lengths of 1471 base pairs, the PacBio system opens up the possibility of identifying microorganisms to the species level in oral cavity (Figure 1).

**Figure 2:**
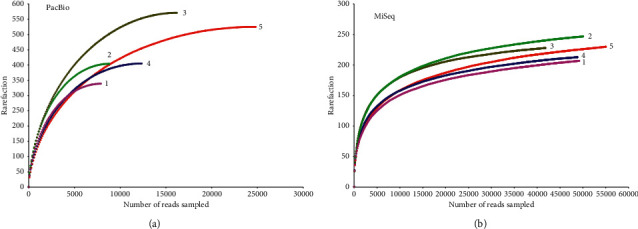
Rarefaction curves for (a) PacBio and (b) MiSeq platforms. The average number of OTUs in each sample was calculated. Samples from the two platforms displayed similar phylogenetic diversity at a 97% identity level.

**Figure 3:**
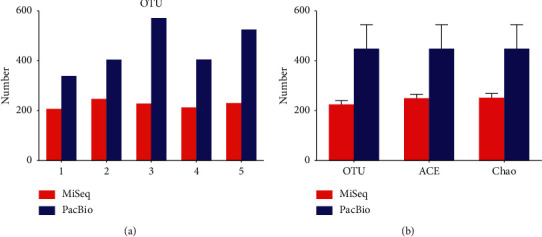
Richness of oral saliva. (a) OTU distribution of the 5 samples sequenced by MiSeq and PacBio platforms. (b) Comparison of OTU number and richness indices (Chao and ACE) between PacBio and MiSeq platforms. Different colors indicate different platforms.

A slightly higher coverage was observed in the PacBio RS II platform, and the level of coverage indicated that the 16S rRNA gene sequences identified by the two sequencing platforms represented the majority of bacterial sequences present in the oral saliva samples. The rarefaction curves and richness indices (Chao and ACE) that estimated the richness of the total oral microbiota also show that enough sequencing data were generated by the two platforms (Figures 2 and 3).

**Figure 4:**
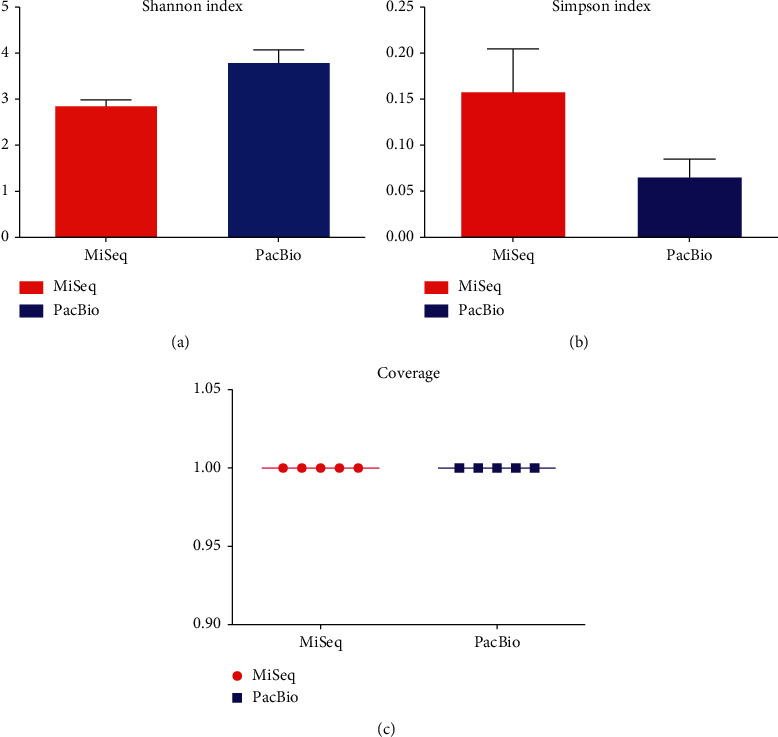
Comparison of *α*-diversity and coverage between MiSeq and PacBio platforms. (a) Shannon index, which can reflect how many OTUs there are in saliva and simultaneously take into account how evenly the OTUs are distributed among the oral microbiome. (b) Simpson index, which is used to measure the degree of concentration when oral microbiota are classified into OTUs. (c) Coverage, which is calculated from the length of the original genome (*G*), the number of reads (*N*), and the average read length (*L*) as *N*∗*L*/*G*.

Clustering the unique sequences into OTUs at 3% dissimilarity resulted in an average of 225 OTUs on the MiSeq platform; however, the number of OTUs generated on the PacBio RS II platform was 449, almost twice as that of the MiSeq platform (Figure 3). Other indices (Chao estimate and Ace index) revealed that the PacBio RS II platform detected more species. The comparisons of alpha-diversity indices (Shannon and Simpson) of the oral microbiota were significantly different between the two platforms. The Shannon index of the MiSeq group was lower than that of the PacBio RS II group, and the Simpson index of the MiSeq group was higher than that of the PacBio RS II group. It was demonstrated that the PacBio RS II platform exhibited a significant higher level of *α*-diversity when compared with the MiSeq platform (Figure 4). In spite of less clean reads, the PacBio RS II system discovered more species than the MiSeq sequencing platform (Figures 2 and 3).

### 3.2. Taxonomic Analysis of Different Platforms

437 species derived from 10 phyla, 17 classes, 24 orders, 31 families, and 60 genera were detected by the PacBio RS II platform, while 163 species derived from 12 phyla, 21 classes, 29 orders, 42 families, and 72 genera were detected by the MiSeq platform.

**Figure 5:**
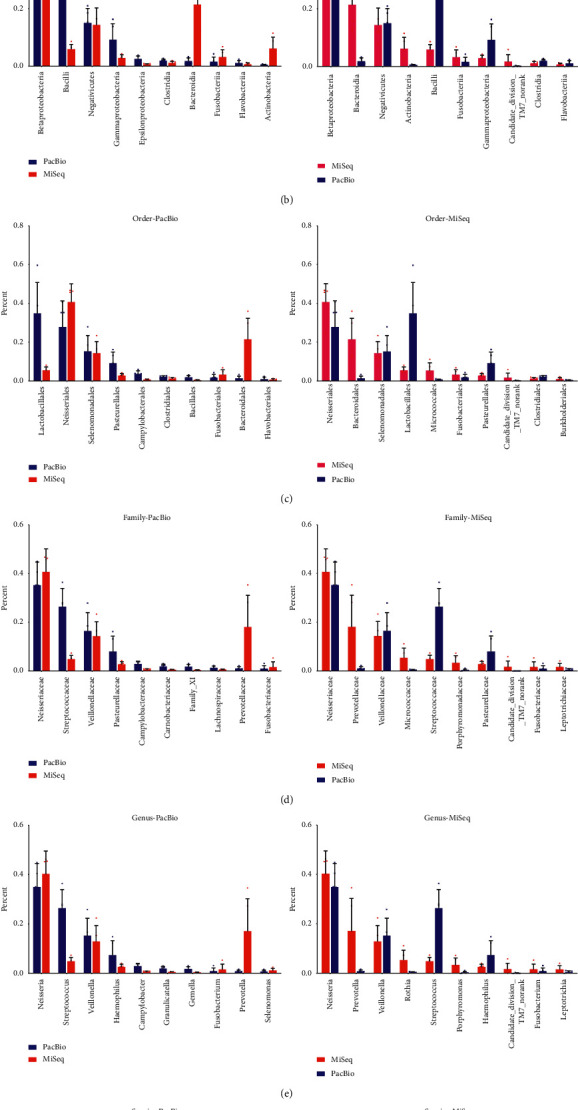
The relative abundance of top 10 phyla, classes, orders, families, and genera and top 15 species. (a) Top 10 phyla. (b) Top 10 classes. (c) Top 10 orders. (d) Top 10 families. (e) Top 10 genera. (f) Top 15 species.

At the phylum level, *Firmicutes*, *Bacteroidetes*, *Proteobacteria*, *Actinobacteria*, *Fusobacteria*, and TM7 shared 95.7% of oral microbiome and 1.17% of oral bacteria cannot be classified by the MiSeq platform. However, on the PacBioRS II platform, *Firmicutes*, *Proteobacteria*, *Bacteroidetes*, *Fusobacteria*, *Actinobacteria*, and TM7 comprised 99.96% of the community and all of the bacteria were annotation to phylum (Figure 5(a)).

**Figure 6:**
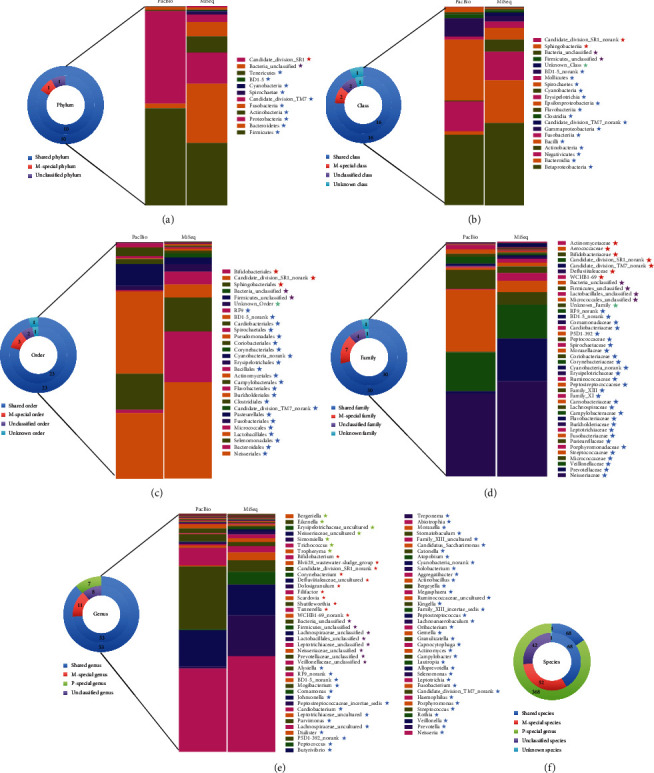
Community structures sequenced by PacBio and MiSeq platforms. 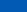 represents the number of organisms shared by the two platforms and the detail taxonomy information was shown on the right bar chart. The star of the same color represents the names of the shared organism. 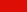 represents the number of organisms generated only by MiSeq platforms and the detail taxonomy information was shown on the right bar chart. The star of the same color represents the names of the organism only generated by MiSeq. 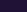 represents the number of unclassified organism and the detail taxonomy information was shown on the right bar chart. The star of the same color represents the names of the unclassified organism. 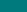 represents the number of unknown organism and the detail taxonomy information was shown on the right bar chart. The star of the same color represents the names of the unknown organism. 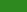 represents the number of organisms generated only by PacBio platforms and the detail taxonomy information was shown on the right bar chart. The star of the same color represents the names of the organism only generated by PacBio. (a–f) represents phylum, class, order, family, genus and species level, respectively.

The overall structure of oral microbiota for each platform at the phylum level is shown in Figure 6(a). Ten phyla were shared by the two platforms, and Candidate_division_SR1 were found only on the MiSeq platform.

At the class level, the majority of the sequences of MiSeq belonged to *Betaproteobacteria*, *Bacteroidia*, *Negativicutes*, *Actinobacteria*, and *Bacilli*, which contributed 93.3% of the whole community. The unknown and unclassified class proportion accounted for 0.79%. For the PacBio platform, *Betaproteobacteria*, *Bacilli*, *Negativicutes*, *Gammaproteobacteria*, and *Epsilonproteobacteria* shared 92.9% of oral microbiome and a minuscule proportion (0.25%) of unknown classes was generated (Figure 5(b)). The overall structure of oral microbiota for each platform at the class level was shown in Figure 6(b). *Betaproteobacteria* accounted for the largest proportion of the total community in both of the two groups, while the abundance of the abundance of Bacilli and Bacteroidia were different between the two platforms.

At the order level, *Neisseriales*, *Bacteroidales*, *Selenomonadales*, *Lactobacillales*, *Fusobacteriales*, *Pasteurellales*, and *Clostridiales* dominated the community in both groups (Figure 5(c)). The overall structure and portion of oral microbiota for each platform were shown in Figure 6(c). The unknown and unclassified order proportion sequencing by MiSeq was 0.79%; however, only 0.25% order was unclassified by the PacBio platform (Figure 6(c)).

At the family level, *Neisseriaceae*, *Prevotellaceaes*, *Veillonellaceae*, *Streptococcaceae*, *Pasteurellaceae*, and *Fusobacteriaceae* shared 82.4% and 88.0% of oral microbiome by the MiSeq and PacBio platforms, respectively (Figure 5(d)). 0.39% and 0.25% of oral bacteria were unknown or cannot be classified by the MiSeq and PacBio platform, respectively (Figure 6(d)).

At the genus level, the majority of the sequences of the two platforms belonged to *Neisseria*, *Prevotella*, *Veillonella*, *Streptococcus*, *Haemophilus*, and *Fusobacterium*, which contributed 79.4% and 86.8% of the MiSeq and PacBio community. The unknown and unclassified genera of the MiSeq platform accounted for 0.68% (Figure 5(e)). The overall structure and portion of oral microbiota for each platform were shown in Figure 6(e).

At the species level, 68 species were shared by the two platforms and 368 species were detected only by the PacBio RS II platform. Forty-two genera cannot be classified into special strains on the MiSeq platform, which accounted for nearly half of the whole community (Figure 6(f)); however, only 0.03% of microorganisms were unidentified when using the PacBio RS II platform.

**Figure 7:**
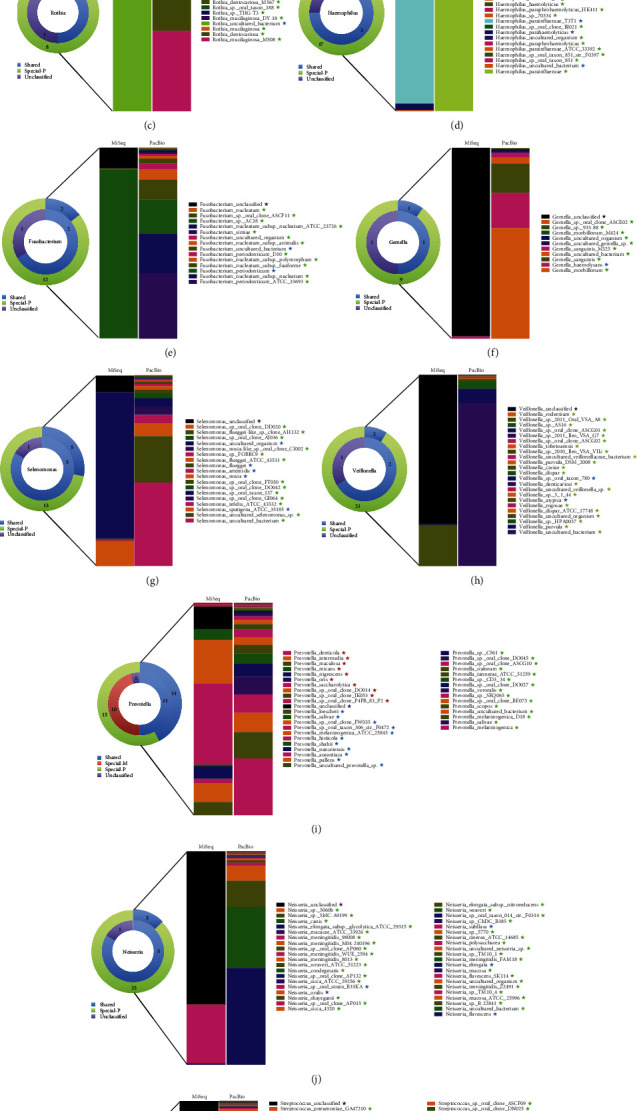
Structure and composition of some particular species sequenced by PacBio and MiSeq platforms. The outer ring of the chart represents the number of species sequenced by the PacBio platform. The inner ring, on the opposite, represents the number of species sequenced by the MiSeq platform. 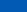 represents the number of species shared by the two platforms, and the detail information was shown on the right bar chart. The star of the same color represents the name of the shared species. 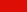 represents the number of species generated only by the MiSeq platform, and the detail information was shown on the right bar chart. The star of the same color represents the name of the species only generated by MiSeq. 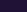 represents the number of unclassified species, and the detail information was marked on the right bar chart. The star of the same color represents the name of the unclassified species. 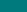 represents the number of species generated only by the PacBio platform, and the detail information was shown on the right bar chart. The star of the same color represents the name of the species only generated by PacBio. (a–k) represents the species of *Actinomyces*, *Campylobacter*, *Rothia*, *Haemophilus*, *Fusobacterium*, *Gemella*, *Selenomonas*, *Veillonella*, *Prevotella*, *Neisseria*, and *Streptococcus*, respectively.

Figure 5(f) shows the top 15 species generated by the two platforms. As is shown in the figure, unlike the other levels, there was a distinction between the most abundant bacteria sequenced by the two platforms. Speculation was that a large proportion of the total bacteria was unclassified by the MiSeq platform. The structure and composition of saliva microbiota shown in Figure 7 lists comparison of some species sequenced by the two platforms. As is shown in Figure 7, unclassified species accounts for a considerable proportion on the MiSeq platform. The PacBio RS II platform, by contrast, had higher resolution and could provide more information at the species level.

For species of *Actinomyces*, 16.2% of the bacteria was unclassified by the MiSeq platform. *Actinomyces odontolyticus* and *Actinomyces uncultured bacterium* were shared by the two platforms, and 7 unique species were generated only by the PacBio RS II platform (Figure 7(a)).

As to the species of *Campylobacter*, 2.2% of the bacteria was unclassified by the MiSeq platform. *Campylobacter concisus* and *Campylobacter showae* were shared by the two platforms, and 8 species were unique to the PacBio RS II platform (Figure 7(b)).

For species of *Rothia*, 10.1% of the bacteria was unclassified by the MiSeq platform. *Rothia uncultured bacterium* was the only species shared by the two platforms, and 8 unique species were generated only by the PacBio RS II platform (Figure 7(c)).

When it comes to *Haemophilus*, 5.7% of the bacteria was unclassified by the MiSeq platform. *Haemophilus parahaemolyticus*, *Haemophilus parainfluenzae T3T1*, and *Haemophilus uncultured bacterium* were shared by the two platforms, and 17 unique species were generated only by the PacBio RS II platform (Figure 7(d)).

For species of *Fusobacterium*, which are among the most abundant bacteria in healthy oral cavity, 10.8% of the bacteria was unclassified by the MiSeq platform. *Fusobacterium periodonticum* and *Fusobacterium uncultured bacterium* were shared by the two platforms, and 12 unique species were generated only by the PacBio RS II platform (Figure 7(e)).

Figure 7(f) shows the composition of *Gemella* sequenced by different platforms. The comparison of sequencing results between MiSeq and PacBio RS II indicates that *Gemella haemolysans* was the only species shared by both the platforms and up to 98.8% species were unclassified by the MiSeq platform. Nine unique species were generated only by the PacBio RS II platform.

For species of *Selenomonas*, 8.6% of the bacteria was unclassified by the MiSeq platform. Five species including *Selenomonas uncultured organism*, *Selenomonas flueggei*, *Selenomonas artemidis*, *Selenomonas noxia*, and *Selenomonas sputigena ATCC 35185* were shared by the two platforms, and 7 unique species were generated only by the PacBio RS II platform (Figure 7(g)).

As to species of *Veillonella*, 78.1% of the bacteria was unclassified by the MiSeq platform. *Veillonella atypica* and *Veillonella* sp. *oral taxon 780* were shared by the two platforms, and 21 unique species were generated only by the PacBio RS II platform (Figure 7(h)).

For species of *Prevotella*, 10.5% of the bacteria was unclassified by the MiSeq platform. 11 species including *Prevotella loescheii*, *Prevotella salivae*, *Prevotella* sp. *oral clone FW035*, *Prevotella* sp. *oral taxon 306 str. F0472*, *Prevotella melaninogenica ATCC 25845*, *Prevotella histicola*, *Prevotella shahii*, *Prevotella nanceiensis*, *Prevotella aurantiaca*, *Prevotella pallens*, and *Prevotella uncultured prevotella* sp. were shared by the two platforms. The number of unique species generated by the MiSeq and PacBio RS II platform were 15 and 11, respectively (Figure 7(i)).

For species of *Neisseria*, which are the most abundant species of the community in this study, 71.7% of the bacteria was unclassified by the MiSeq platform. Five species including *Neisseria* sp. *oral strain B33KA*, *Neisseria oralis*, *Neisseria subflava*, *Neisseria elongata*, and *Neisseria flavescens* were shared by the two platforms. The number of unique species generated by PacBio RS II platform was up to 35 (Figure 7(j)).

*Streptococcus* is a gram-positive bacterium belonging to the phylum *Firmicutes*, which is found to be associated with many kinds of oral diseases, such as caries [18, 27], pneumonia, bacteremia, and meningitis [28, 29]. In this study, 73.4% of *Streptococcus* was unclassified by the MiSeq platform. Only the two species *Streptococcus intermedius* and *Streptococcus sanguinis* were shared by the two platforms. The number of unique species generated by the PacBio RS II platform was up to 77 (Figure 7(k)).

## 4. Discussion

A number of research studies have presented evidence for using childhood oral microbiome to predict future oral and systemic diseases [30]. Therefore, it is very important for us to find a suitable sequencing method to study oral microbiome. In this study, the oral saliva microbiome of five healthy Chinese children was evaluated using the NGS and TGS. The oral microbiome composition sequenced by the two platforms was basically identical from phylum to genus level. The structure of oral microbiome at the species level, however, showed a significant difference between the two platforms. The possible reason we speculate is that a large amount of short reads generated by the MiSeq platform cannot be resolved in spite of the development of the assemblers, such as the Celera Assembler, SOAPdenovo, and Allpath-LG. As a result, a very large proportion of bacteria was unclassified by the MiSeq sequencing technology. The longer reads sequenced on the PacBio platform gave more phylogenetic resolution than 400–500 bp fragments that contain fewer hypervariable regions.

Compared with our previous study on the structure of oral microbiome in healthy children, the top 10 phyla, genera, and species are consistent [18]. However, when compared with other studies, there are some differences with our results [31]. In this respect, we speculated that oral microbiome is linked to age, race, and region at the species level. Some studies have also demonstrated that the oral microbiota are better defined based on age, gender, oral niches, and even the body size [32, 33]. Recent findings indicate that the oral ecosystem of healthy children is highly heterogeneous and dynamic with substantial changes in microbial composition over time and only few taxa persisting across the age [34]. PacBio RS II sequencing, one platform of TGS, has the ability to provide longer sequences and reads generated from sequencing the entire 16S rRNA gene. Compared with the previous NGS, this platform can establish a higher estimate of richness and provide the ability to identify organisms at a higher taxonomic and phylogenetic resolution [17, 18, 35]. At the same time, some studies have shown that the PacBio sequencing error rate is in the same range of the previously widely used Roche 454 sequencing platform and the current MiSeq platform [36, 37]. More importantly, a recent study presented a high-throughput amplicon sequencing methodology based on PacBio CCS that measures the full-length 16S rRNA gene with a near-zero error rate [38].

Compared with the traditional 16S rDNA sequencing of the MiSeq platform, the PacBio RS II technology improved its read length and annotated the nucleotide sequence of oral bacteria to the species level. PacBio RS II may be optimal for oral microbiome sequencing due to its long reads and high performance, while platforms such as Illumina MiSeq will provide cost-efficient methods for sequencing projects.

Previous research studies had compared the TGS PacBio platform with the NGS Roche 454 pyrosequencing platform. Amplicons of the 16S rRNA gene from the environmental samples from streambed habitats, rocks, sediments, and a riparian zone soil were analyzed [16, 17]. In this study, we focus on the oral microbiome of healthy Chinese children and compare the amplicons of the 16S rRNA gene between PacBio and MiSeq platforms. As the exact composition of the microbiome from the five Chinese children were unknown, it is still difficult to assess the accuracy of the PacBio RS II platform at the species level. Next, we would enroll a known isolate as a positive control in high-throughput sequencing, which can provide the quality assurance of quantifying error rates when analyzing environmental communities.

## 5. Conclusions

In our study, oral microbiome of healthy Chinese children was explored. For oral microbiome studies, if the goal is identifying all species in a sample, PacBio appears to have superior performance to MiSeq. However, if the goal is to simply quantify relative differences in diversity, either platform would be appropriate. In this article, we have compared the difference between the two platforms, however, with the limited sample size, the study does not provide a statistic conclusion, and more in-depth studies with larger group sizes are needed to validate these results.

## Data Availability

The data sets used and/or analyzed during the current study available from the corresponding author on reasonable request. The authors have deposited the raw data and clean data (after QC and human reads removal) to the NCBI under accession number PRJNA445629.
